# Synaptogenesis by Cholinergic Stimulation of Astrocytes

**DOI:** 10.21203/rs.3.rs-2566078/v1

**Published:** 2023-02-13

**Authors:** Pamela J. Roqué, Andrés Barria, XIAOLU ZHANG, Lucio G. Costa, Marina Guizzetti

**Affiliations:** University of Washington; University of Washington; Oregon Health and Science University; University of Washington; Oregon Health and Science University

**Keywords:** acetylcholine, astrocytes, hippocampal neurons, TSP1, PSD-95, synaptophysin, synaptogenesis

## Abstract

Astrocytes release numerous factors known to contribute to the process of synaptogenesis, yet knowledge about the signals that control their release is limited. We hypothesized that neuron-derived signals stimulate astrocytes, which respond by signaling back to neurons through the modulation of astrocyte-released synaptogenic factors. Here we investigate the effect of cholinergic stimulation of astrocytes on synaptogenesis in co-cultured neurons. Using a culture system where primary rat astrocytes and primary rat neurons are first grown separately allowed us to independently manipulate astrocyte cholinergic signaling. Subsequent co-culture of pre-stimulated astrocytes with naïve neurons enabled us to assess how prior stimulation of astrocyte acetylcholine receptors uniquely modulates neuronal synapse formation. Pre-treatment of astrocytes with the acetylcholine receptor agonist carbachol increased the expression of synaptic proteins, the number of pre- and postsynaptic puncta, and the number of functional synapses in hippocampal neurons after 24 hours in co-culture. Astrocyte secretion of the synaptogenic protein thrombospondin-1 increased after cholinergic stimulation and the inhibition of the target receptor for thrombospondins prevented the observed increase in neuronal synaptic structures. Thus, we identified a novel mechanism of neuron-astrocyte-neuron communication, *i.e*., neuronal release of acetylcholine stimulates astrocytes to release synaptogenic proteins leading to increased synaptogenesis in neurons. This study provides new insights into the role of neurotransmitter receptors in developing astrocytes and into our understanding of the modulation of astrocyte-induced synaptogenesis.

## Introduction

Astrocytes are actively involved in neuronal development, as they participate in neuritogenesis, neurite outgrowth [1, 2], and synaptogenesis [3–8]. Astrocyte-released cholesterol, thrombospondin-1 and 2 (TSP1/TSP2), high endothelial venule protein (hevin), TGF-β and TGF-β1, glypicans 4 and 6, chordin-like 1 (Chdrl1), and pentraxin 3 (PTX3) are among the astrocyte-released/expressed factors that enhance astrocyte-induced glutamatergic, cholinergic or GABAergic synaptogenesis. Each of these factors have different effects, acting either during the period of synaptic initiation in early synaptogenesis, or influencing synaptic maturation to functional synapses by clustering and insertion of α-amino-3-hydroxy-5-methyl-4-isoxazoleprionic acid (AMPA) receptors at the surface of postsynaptic receptors [3, 6, 9–18]. Additionally, astrocyte released secreted protein acidic and rich in cysteine (SPARC) inhibits the synaptogenic effect of hevin [16]. Despite the increasing identification of astrocyte-released synaptogenic factors, knowledge of the signals controlling their release remains for the most part unknown.

With processes that extend into the synaptic space, astrocytes are in close proximity to neuronal synapses [19] making them ideally located to respond to environmental cues from neighboring neurons [20]. In fact, they express most neurotransmitter receptors [21–24] and are capable of responding to synaptic neurotransmitter release [25–28], including glutamate [29, 30], ACh [25, 31, 32], γ-aminobutyric acid (GABA) [33], adenosine triphosphate (ATP) [34], and endocannabinoids [35–37].

The neurotransmitter acetylcholine (ACh) has been shown to influence various aspects of early brain development, including neuronal and glial proliferation, and neuronal differentiation and migration [38–42]. The cholinergic system appears early in development and spontaneous cholinergic activity influences synaptogenesis [43–45]. Interestingly, astrocytes, which proliferate during the time in development when synapses are forming [8, 46, 47], are capable of responding to acetylcholine with calcium elevations [25, 26]. In addition, cholinergically-induced synaptic plasticity has been shown to be mediated by astrocytes [31, 32].

The fact that astrocytes are integral to neuronal development and that they express ACh receptors (AChRs) and respond to synaptic release of ACh suggests that stimulation of astrocyte AChRs may promote neuronal development by modulating the expression and release of factors that create an environment conducive to it. Here we test the hypothesis that the selective cholinergic stimulation of astrocytes increases synaptogenesis through the release, by astrocytes, of factors that potentiate the formation of synaptic structures in co-cultured neurons. Our results show that pre-treatment of astrocyte cultures with a cholinergic agonist leads to enhanced expression of neuronal synaptic proteins, an increase in the number of pre- and postsynaptic puncta, and an increase in the number of structural and functional synapses in hippocampal neurons co-cultured with carbachol-pre-treated astrocytes. This enhanced synaptogenic function of astrocytes is prevented by the inhibition of TSP receptors in the co-culture system and mimicked by exogenous TSP1, an extracellular matrix protein whose release is increased after cholinergic stimulation of astrocytes. The elucidation of the signals that regulate changes in astrocyte-secreted proteins involved in synaptogenesis is a key step toward the understanding of normal and pathological neurodevelopmental processes.

## Materials And Methods

### Materials

Neurobasal-A medium, Dulbecco’s Modified Eagle’s Medium, B-27 Supplement, GlutaMax, fungizone and gentamicin were purchased from Gibco. Poly-l-ornithine hydrobromide (PLO), poly-d-lysine (PDL), cytosine d-arabinoside (ARAC), carbamylcholine chloride (carbachol), mecamylamine hydrochloride, gallamine triethiodide, and gabapentin were purchased from Sigma-Aldrich. 1,1 Dimethyl-4-diphenylacetoxypiperidinium iodide (4-DAMP) was purchased from Tocris Biosciences. Rabbit monocolonal antibody to synaptophysin (ab52636) and rabbit polyclonal antibody to synaptotagmin (ab10104) were purchased from Abcam. The mouse monoclonal antibody to PSD95 (7E3–1B8) used in immunocytochemistry was purchased from Thermo Scientific. The mouse monoclonal antibodies PSD95 (Clone K28/43) for immunoblotting, and NR2B (Clone N59–36) were developed by and obtained from the UC Davis/NIH Neuromab facility. The goat anti-mouse TSP1 antibody (A6.1) was obtained from EMD/Millipore/Calbiochem. Donkey anti-rabbit Alexa Fluor 488 and donkey anti-mouse Alexa 555 were obtained from Invitrogen. Anti-mouse horseradish peroxidase (HRP) was purchased from BD Biosciences and anti-rabbit HRP was obtained from Cell Signaling Technology. Human TSP1 was obtained from Haematologic Technologies, Inc.

### Hippocampal Neuron Preparation and Culture

Primary hippocampal neurons from E21 Sprague Dawley rats were prepared as previously described [48, 49]. Briefly, cells were plated in poly l-ornithine hydrobromide (PLO,15 μg/mL) pre-coated 6-well plates (1.5 × 10^6^/well) for protein expression experiments, or on PLO pre-coated glass coverslips (12 mm circles) with manually adhered paraffin spacers for co-culture experiments (0.08 × 10^6^ neurons/coverslip) placed into 24 well plates (1 coverslip/well). Neurons were maintained in Neurobasal-A medium supplemented with B-27 neuronal survival and growth factors (1%), 3 mM GlutaMax, 30 mM D- (+) glucose solution, 0.5% fungizone, and 100 μg/mL gentamicin and maintained at 37°C for 12–13 days prior to co-culture. To hinder astrocyte proliferation, ARAC was added at a final concentration of 2.5 μM to each well after three days in culture. One-third of the medium was changed every three to four days post ARAC treatment. On day in culture (DIC) 12–13, neurons were either co-cultured with primary astrocytes or treated with human TSP1 for 24 hours (see below).

### Primary Astrocyte Preparation and Culture

Primary cortical astrocytes were dissected and prepared from E21 Sprague Dawley rat pups, as previously described [39, 48, 49] and cultured in 75 cm^2^ flasks coated with 40 μg/mL PDL at an initial density of 2.5–3.0 × 10^6^ cells/flask. Astrocytes were grown to confluency in Dulbecco’s Modified Eagle’s Medium supplemented with 10% fetal bovine serum and penicillin (100 U/mL) and streptomycin (100μg/mL) (P/S).

### Astrocyte Treatments and Neuronal Co-Culture

Primary cortical astrocytes were trypsinized from established cultures and sub-cultured in 24 well plates (250,000 cells/mL) for synaptogenesis and electrophysiology experiments, or on the underside of 0.4 μm mesh 6-well plate inserts for protein expression experiments. Wells and inserts were coated with 40 μg/mL PDL. Forty-eight hours prior to co-culture with neurons, astrocytes were serum deprived (DMEM + 0.1% bovine serum albumin and P/S) for 24 hours, and then treated with or without carbachol (0.01, 0.10, 1 mM) for an additional 24 hours. Astrocytes were washed with PBS and incubated in serum-free medium for three hours prior to co-culture with neurons. In some experiments, 30 minutes prior to the addition of carbachol, astrocytes were treated with 10 μM of the acetylcholine receptor antagonists, mecamylamine, gallamine, or 4-DAMP. After treatment washout and medium conditioning, primary hippocampal neurons (12–13 DIC), grown on glass coverslips were inverted over the pre-treated astrocyte monolayer for immunocytochemistry or electrophysiology experiments. Neurons were never in direct contact with astrocytes, nor were they exposed to astrocyte treatments. For Western blot experiments, astrocytes plated on the underside of porous inserts and their medium were transferred to 6-well plates containing primary hippocampal neurons.

To block TSP1 signaling in neurons, after astrocyte carbachol pre-treatment and washout, neurons and astrocytes were pre-treated for half hour with gabapentin (15 or 30 μM) prior to co-culturing; gabapentin remained throughout the co-culture incubation. For all co-culture experiments, astrocytes and neurons were co-incubated for 24 hours.

### Immunocytochemistry, Image Acquisition and Analysis

Neurons were fixed in 4% PFA for 20 minutes at 37°C, blocked and permeabilized for 30 minutes (0.1% Triton-X, 5% bovine serum albumin in PBS) and co-labeled with primary antibodies against synaptophysin (1:250) and PSD-95 (1:200), or synaptotagmin (1:250) and NR2B (1:200) for at least 18 hours at 4°C under slow rocking. Coverslips were then incubated for 1 hour at room temperature with fluorescent secondary donkey anti-rabbit Alexa 488, and donkey anti-mouse Alexa 555 antibodies (1:500) and the nuclear dye Hoechst 33342 (1 μg/mL). Coverslips were mounted on glass slides using Vectashield, topped with a cover glass, and sealed with nail polish. Individual, healthy neurons, identified with Hoechst stain, and located at least two cell bodies from their nearest neighbor, were imaged using confocal microscopy (Olympus Fluoview-1000) Confocal images were acquired using a 60X oil immersion objective, a 1024 × 1024 image size with a 2X optical zoom, yielding a 0.103 μm/pixel resolution. An average of 12–18 slices per neuron were acquired through the z-plane, using a step size of 0.30 μm from 5 neurons per coverslip. To capture the entire range of fluorescent signal, the slide from the treatment group with the greatest intensity was used to set the imaging parameters for each channel and the settings remained constant during acquisition of images from each treatment groups within an experiment. Images were deconvolved using Huygens Professional software (Scientific Volume Imaging). For object analysis, the surface of synaptic puncta was rendered three-dimensionally based on a size and intensity threshold which was determined by obtaining the mean intensity of 30 manually selected, size appropriate puncta from 15 neuronal fields of each channel (350–450 total) using Image J, from the treatment group used to set confocal imaging parameters. Channel thresholds were held constant for all treatment groups within an experiment. The number of individual pre and postsynaptic objects and those overlapping between channels were automatically calculated and recorded using Huygens Object Analysis software.

For immunocytochemical labeling of TSP1 on the astrocyte surface, astrocytes were plated on PDL-coated glass coverslips at a density of 250,000 per well, in 24 well plates. Control and carbachol-treated astrocytes were incubated for 24-hours, washed in PBS, and fixed in 4% PFA for 20 minutes at 36°C, but not permeabilized in order to label only cell-surface TSP1. Astrocytes were then blocked in 10% goat serum and incubated in anti-mouse TSP1 primary antibody (1:80 in PBS) for 3 hours at room temperature, followed by donkey anti-mouse Alexa 488 (1:75, PBS, 3% BSA) secondary antibody and the nuclei stained with Hoechst 33342 (1:2000 in PBS). Cells were mounted on slides using Vectashield and imaged using confocal imaging with parameters held constant between treatment groups. Confocal images were acquired using a 40X oil immersion lens, at a step-size of 0.50 μm for a total of 18–21 planes per field. Nine to ten fields were imaged per treatment group from two coverslips. For each field, the integrated optical density per plane was determined using Metamorph software (Molecular Devices), totaled, normalized to cell number and averaged for each treatment group. For presentation of a representative image, the maximum intensity Z-projection was obtained from confocal images using Image J, and channels were merged.

### Western blot

Neurons were lysed in 1% SDS lysis buffer, sonicated twice at 3.5 power for 5 seconds and the protein quantified using the bicinchoninic acid assay (BCA). Equal amounts of protein were loaded into freshly cast bis-trisacrylamide gels (10%) and proteins were separated by gel electrophoresis then transferred to PVDF membranes. Membranes were blocked in TBST (5% milk) for 1 hour, then probed overnight at 4°C with primary antibodies for synaptophysin (1:500), or PSD-95 (Neuromab, 1:500). Membranes were incubated for 1 hour with HRP-conjugated secondary antibodies (1:1000) and developed. Band densitometry was determined using Image J software, and results normalized to total protein as determined by Coomassie blue protein stain.

For measurement of astrocyte TSP1 levels, astrocytes were plated on PDL (40 μg/mL) pre-coated 100 mm plates at a density of 2.5 × 10^6^ per plate and cultured for 4 days, serum-deprived for 24 hours, then treated for 24 hours with carbachol (1 mM) prepared in serum free medium. Medium was collected and cells were lysed in 1% SDS lysis buffer from treated and untreated plates immediately after the 24h treatment (time 0), and 6 and 24 hours after treatment washout. Intracellular protein was quantified from lysate samples using the BCA method and equal amounts were loaded into 3–8% Tris-Acetate gels and separated by gel electrophoresis. After transfer, PDVF membranes were blocked in TBST (3% BSA) for 1 hour and probed overnight in goat anti-mouse TSP1 (1:1000), then incubated for 1 hour with HRP-conjugated secondary antibodies (1:1000) and developed. TSP1 was detected by chemiluminescence and normalized to β-actin levels using densitometric analysis. For measurements of TSP1 in the astrocytic medium, 7 mL of media from each time point was concentrated to 200 μl using Pierce concentrators (MWCO 9kDa) centrifuged at 4000 × g for 25 minutes at 25 C. After the addition of protease inhibitors, reducing reagents, and sample buffer, samples were denatured by heating (70°C for 10 minutes) and equal volumes (30 μl) of concentrated sample were loaded into 3–8% Tris-Acetate pre-cast gels. After transfer, PDVF membranes were blocked in TBST (3% BSA) for 1–3 hours and incubated overnight in goat anti-mouse TSP1 (1:500). Band densitometry was determined using Image J software and normalized to corresponding cell lysate protein concentrations.

### Electrophysiology

Spontaneous miniature excitatory postsynaptic currents (mEPSCs) were recorded at room temperature (21.5–23.5°C) from hippocampal neurons after co-culture with carbachol (1 mM) pre-treated astrocytes, or untreated astrocyte controls using whole cell patch clamp techniques. Currents were recorded using a MultiClamp 700B by Axon Instruments; cells were voltage clamped at a holding potential of −70 mV. Patch pipettes were pulled from borosilicate capillary glass (2.4–5.9 M). Dissociated hippocampal neurons were bathed in artificial cerebral spinal fluid (ACSF) (119 mM NaCl, Ω 2.5 mM KCl, 4 mM CaCl2, 4 mM MgCl2, 26 mM NaHCO3, 1 mM NaH2PO4, 11 mM glucose at pH of 7.4 and gassed with 5% CO2 and 95% O2). Tetrodotoxin (1 uM) was added to the circulating bath to isolate spontaneous miniature postsynaptic currents. Internal solution contained: 115 mM CsMeSO_4_, 20 mM CsCl, 2.5 mM MgCl_2_, 10 mM HEPES, 4 mM Na_2_ATP, 0.4 mM Na_3_GTP, 10 mM Na-phosphocreatine, and-0.6 mM EGTA, pH 7.25 (CsOH). Activity was recorded for up to ten minutes and events were manually selected using Synaptosoft Mini Analysis software. Traces from individual neurons of each treatment group were combined into arrays and the inter-event interval (IEI), amplitude, and time to decay, cumulative distributions were plotted (7–9 neurons per treatment group array from 3 independent experiments). The frequency of events, as measured by inter-event-intervals, was used to represent the relative number of functional synapses.

### Statistical Analysis

Student’s t-test was used for statistical comparison of the means in experiments containing two treatment groups and One-way ANOVA with Dunnett’s Multiple Comparison test was used where multiple concentrations were assessed relative to control. To compare multiple treatments, one-way ANOVA followed by Bonferroni’s Multiple Comparison tests was performed. Electrophysiology analysis was performed using Synaptosoft Mini Analysis software and Graph Pad Prism. Kolmogorov-Smirnov Two Sample Analysis was used to compare the cumulative distributions from arrayed electrophysiology recordings. In figures where individual data points are presented using dot plots, the means and standard error of the means (SEM) are shown. For experiments with a high number of data points, the distribution of the data, the median, and the interquartile range, or the 25th and 75th percentiles, are presented using violin plots and significant differences of the means are indicated.

## Results

### Cholinergic-stimulation of astrocytes enhances astrocyte-induced synapse formation

To test whether cholinergic stimulation of astrocytes affects synapse formation we treated astrocyte cultures for 24 hours with increasing concentrations of the acetylcholine receptor (AChR) agonist carbachol (0.01, 0.1, or 1 mM). At treatment end, carbachol was removed and fresh treatment-free medium was added. Primary hippocampal neurons, grown separately on glass coverslips, were then inverted over the pre-treated astrocytes for an additional 24 hours. In this co-culture system neurons were neither directly exposed to carbachol nor were they in contact with the astrocytes, limiting any effect on neuronal synapse formation to the modulation of astrocyte-secreted factors.

**Figure 1:**
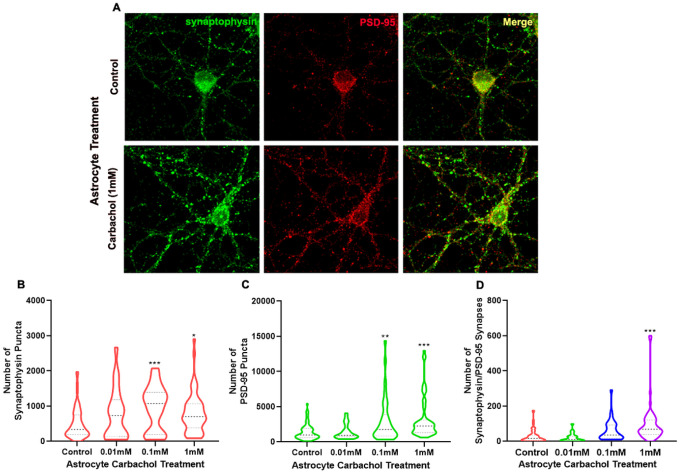
Cholinergic stimulation of astrocytes potentiates synaptic structure formation. Astrocytes were treated with increasing concentrations of carbachol (0, 0.01, 0.10, and 1 mM) for 24 hours. After treatment washout, primary hippocampal neurons, grown on glass coverslips, were inverted over astrocytes and co-cultured for 24 hours. Neurons were immunocytochemically labeled for the pre and postsynaptic proteins synaptophysin and PSD-95 and imaged using confocal microscopy. Synaptic structures were assessed using three-dimensional object analysis. (**A**) Representative deconvolved images of hippocampal neurons after co-culture for 24 hours with astrocytes pretreated with 1 mM carbachol or with control astrocytes. Synaptophysin is shown in green pseudocolor; PSD-95 is shown in red; overlapping puncta are shown in yellow. Quantification of the number of synaptophysin puncta (**B**), PSD-95 puncta (**C**), and overlapping puncta (**D**) in neurons exposed for 24 hours to control astrocytes or astrocytes pre-treated with increasing concentrations of carbachol. Control and 1 mM treatments: n= 55–66 neurons from 6 independent experiments; 0.010 and 0.100 mM treatments: n = 21–34 neurons from 3 independent experiments. The bold, dashed line represents the median; the upper and lower dotted lines represent the 75^th^ and 25^th^ percentile, respectively. One-way ANOVA followed by Dunnett’s Multiple Comparison test was used for statistical comparison of the means. *: p < 0.05, **: p < 0.01, ***: p < 0.001 *vs* control.

To quantify the effect of astrocyte cholinergic stimulation on the localization of synaptic proteins into excitatory synaptic structures, we generated three-dimensional surface renderings of the clustered synaptophysin and PSD95 protein puncta from deconvolved confocal images and the number of pre and postsynaptic specializations were automatically counted for each neuronal field. Astrocytes pre-treated with carbachol increased the number of individual presynaptic synaptophysin (Fig. 1B) and postsynaptic PSD-95 (Fig. 1C) puncta in neurons in a dose-dependent manner. An almost two-fold increase in synaptophysin puncta and a 2.2-fold increase in PSD-95 puncta were observed in neurons after co-culture with astrocytes pre-treated with 0.1 mM carbachol, when compared to control astrocytes. Astrocyte pre-treatment with 1mM carbachol induced a 1.6- and 2.3-fold increase in the number of individual synaptophysin and PSD-95 puncta, respectively (Fig. 1B, C). Because synapses are structures consisting of aligned pre and postsynaptic specializations, we used the number of overlapping synaptophysin and PSD-95 puncta as a measure of the number of structural synapses in each treatment group. Stimulation of astrocytes with carbachol induced the formation of a greater number of synapses than those induced by untreated astrocytes. Pre-treatment of astrocytes with 1 mM carbachol caused a 3.2-fold increase in the number of aligned pre and postsynaptic puncta (structural synapses) above the number of synapses observed when neurons were co-cultured with unstimulated astrocytes (Fig. 1D). Representative deconvolved images of hippocampal neurons after co-culture for 24 hours with astrocytes pretreated with 1 mM carbachol or control astrocytes are shown in Fig. 1A with synaptophysin shown in green, PSD-95 shown in red, and the overlapping puncta shown in yellow. Together, these results indicate that astrocyte-induced hippocampal synaptic structures are greatly increased when neurons are co-cultured in the presence of astrocytes previously stimulated with the cholinergic agonist carbachol.

**Figure 2:**
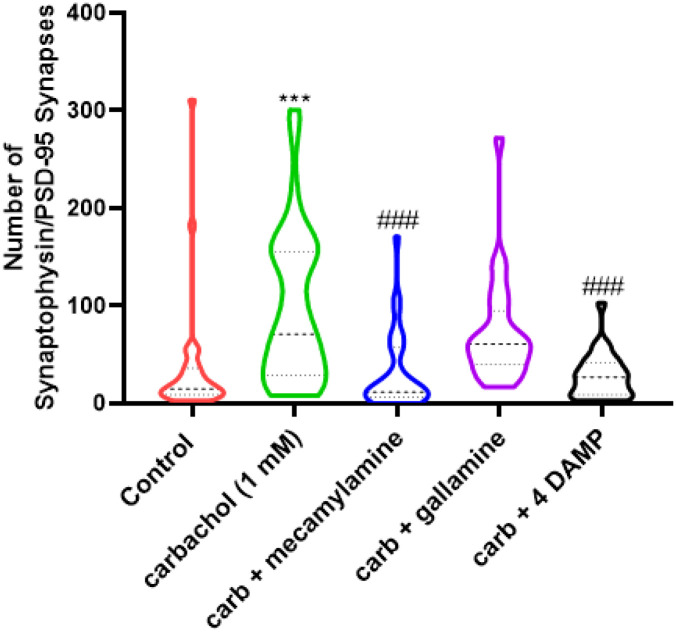
The inhibition of M3 muscarinic and nicotinic receptors in astrocytes reduces carbachol-stimulated astrocyte-induced increase in neuronal synaptic structure formation. Astrocytes were incubated with 1 mM carbachol in the presence or absence of nicotinic receptor antagonist mecamylamine, M2-muscarinic receptor antagonist gallamine, and M3-muscarinic antagonist 4-DAMP (10 μM each) for 24 hours. After treatment washout, hippocampal neurons were co-cultured with astrocytes for 24 hours and synaptic structures, i.e., overlapping synaptophysin and PSD-95 puncta, were quantified. The distribution of the data is shown. The bold, dashed line represents the median; the upper and lower dotted lines represent the 75^th^ and 25^th^ percentile, respectively; n= 25–33 neurons from 3 independent experiments. Statistical comparison of the means was carried out by one-way ANOVA followed by Bonferroni’s Multiple Comparison test. ***: p < 0.001 vs. control; ###: p < 0.001 vs. carbachol.

To determine the cholinergic receptor subtype(s) involved in carbachol-stimulated astrocyte-induced synaptogenesis, astrocyte cultures were pre-treated with carbachol (1 mM) in the presence of muscarinic and nicotinic AChR antagonists for 24 hours before co-culturing the astrocytes with hippocampal neurons. As rat cortical astrocytes do not express M1 or M4 muscarinic receptors [39], we investigated the role of M2 and M3 muscarinic receptors. Inhibiting the M2 muscarinic AChR with gallamine (10 μM) had no effect on carbachol-induced increase in synapse number, while the M3 muscarinic antagonist 4-DAMP reduced the increase in the number of structural synapses observed (Fig. 2). The effect of carbachol-treated astrocytes on synapse formation was also inhibited by the nicotinic antagonist mecamylamine (10 μM) (Fig. 2). These data suggest that cholinergic signaling through both astrocytic M3 muscarinic and nicotinic receptors were responsible for the observed increase in hippocampal neuron synapse number.

**Figure 3:**
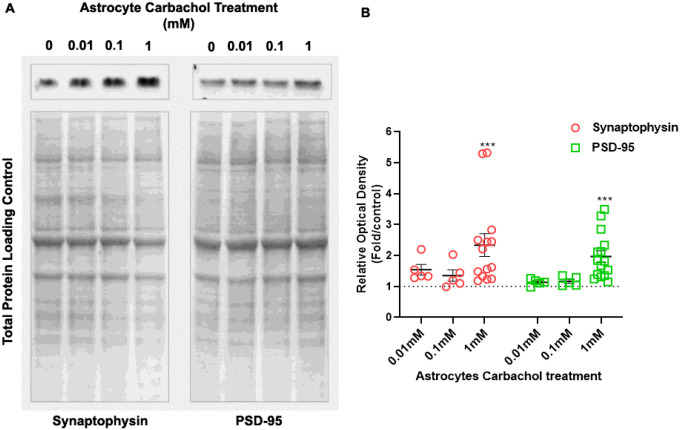
Snaptophysin and PSD-95 protein levels increase in hippocampal neurons after co-culture with carbachol-stimulated astrocytes. Hippocampal neurons plated in glass coverslips were co-cultured for 24 hours with astrocytes plated in 24-well plates and pre-stimulated with increasing concentrations of carbachol (0, 0.01, 0.10, and 1 mM). At the end incubations, protein from neuronal cultures were extracted and Western blots were run for synaptophysin and PSD-95; synaptophysin and PSD-95 protein levels were normalized to total protein content determined after Coomassie blue stain of the membranes. (A) Representative immunoblots for synaptophysin (upper left) and PSD-95 (upper right); lower images: representative loading control membranes after Coomassie blue staining. (B)Densitometric quantification of synaptophysin and PSD-95 bands normalized to total protein content and expressed as fold over control. Control is represented as dotted line. Shown are means ± SEM. Statistical analysis was performed using one-way ANOVA followed by a Dunnett’s Multiple Comparison test (***: p < 0.001); n = 5 coverslips for 10 and 100μM carbachol; n = 14–15 coverslips for control and 1 mM carbachol.

Using Western blotting techniques, we examined total expression of the pre- and postsynaptic proteins synaptophysin and PSD-95. Neurons co-cultured with 1 mM carbachol pre-treated astrocytes showed a robust increase in synaptophysin and PSD-95 protein levels relative to control (Fig. 3A, B). These findings suggest that the increase in puncta size and intensity may be due, at least in part, to increased expression of neuronal synaptophysin and PSD95 expression in neurons.

**Figure 4:**
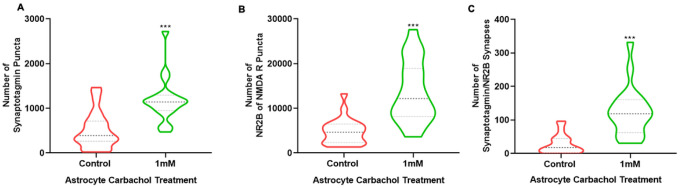
Carbachol-stimulated astrocytes increase the number of synaptotagmin and NR2B pre and postsynaptic puncta and their overlap. Hippocampal neurons were co-cultured for 24 hours with astrocytes pre-treated with 1mM carbachol. Neurons were immunolabeled for the presynaptic protein synaptotagmin and the postsynaptic protein NR2B. Synaptotagmin (**A**) and NR2B (**B**) puncta and their overlap (**C**) were quantified after confocal imaging using three-dimensional object analysis. The bold, dashed line represents the median; the upper and lower dotted lines represent the 75^th^ and 25^th^ percentile, respectively; n = 19–24 neurons from 6 coverslips. Statistical comparison of the means was performed using the Student’s t-test. ***: p < 0.001 vs. control.

We also immunolabeled neurons with two other markers of synaptic structures, the presynaptic protein synaptotagmin, and the postsynaptic NMDA receptor NR2B subunit. We observed a 2.3-fold increase in synaptotagmin puncta (Fig. 4A), a 2.8-fold increase in the NR2B puncta (Fig. 4B), and a 4.6-fold increase in synaptotagmin/NR2B overlapping puncta (Fig. 4C) in neurons co-cultured with carbachol-pre-treated astrocytes, compared to untreated astrocytes. Together this data indicates that cholinergic stimulation of astrocytes enhances synapse formation in co-cultured hippocampal neurons.

### Cholinergic stimulation of astrocytes increases the number of functional synapses

**Figure 5:**
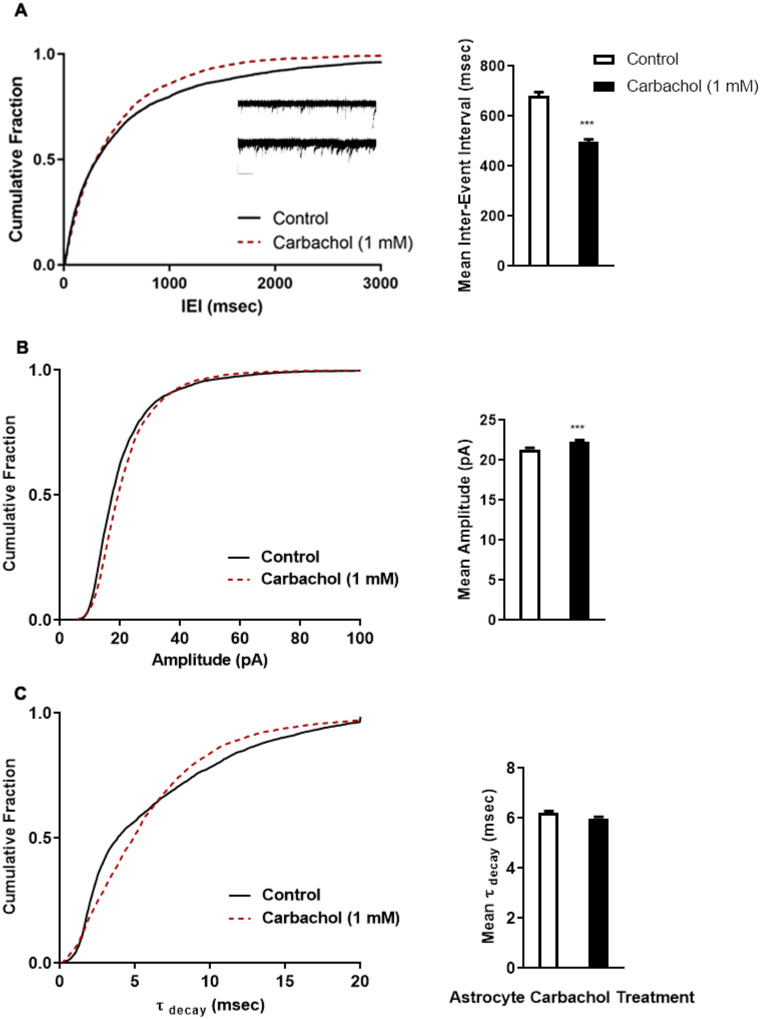
Neurons co-cultured with carbachol pre-treated astrocytes have a higher frequency of mEPSC events. Whole cell patch clamp techniques were used to record spontaneous, miniature excitatory postsynaptic currents (mEPSCs) from dissociated hippocampal neurons after 24 hours of co-culture with carbachol pre-treated or untreated astrocytes. Cumulative distributions of arrayed spontaneous mEPSC inter-event interval (IEI) (**A**), amplitude (**B**) and time to decay (**C**) are shown on the left. **Inset A:** Example traces of neurons co-cultured with control (top) and carbachol pre-treated astrocytes (bottom) (Scale bar: 20 pA, 1 sec). Graphs of the mean IEI (**A**), amplitude (**B**), and time to half decay (C) from each array are shown on the right. Control array n = 9, carb array n= 7 neurons from 3 independent experiments. Cumulative distributions of IEI, amplitude, and time to half decay differ significantly (all < 0.0001) as measured by the Kolmogorov-Smirnov Two Sample Test (IEI: K-S D = 0.0714; amplitude: K-S D = 0.1048; decay time: K-S D = 0.1215). Student’s t-test was performed to compare the means of the arrayed data per treatment group. ***: p < 0.0001 vs control.

We measured the frequency of spontaneous miniature excitatory postsynaptic currents (mEPSCs) [50] in neurons co-cultured with astrocytes pre-treated with carbachol compared to neurons co-cultured with control astrocytes and found a significantly greater frequency of mEPSCs, as measured by the mEPSCs inter-event interval (Fig. 5A), indicating a greater number of functional synapses in neurons co-cultured with carbachol-stimulated astrocytes. A slight, but statistically significant increase in the amplitude was also observed (Fig. 5B), suggesting a potential increase in the number of postsynaptic receptors in neurons co-cultured with carbachol astrocytes. Additionally, although there was no difference in the mean time to half decay between treatment groups, neurons co-cultured with control astrocytes relative to carbachol pre-treated astrocytes, showed faster receptor kinetics in a proportion of the synapses, but slower kinetics in the remainder (Fig. 5C). This observation suggests potential differences in the composition of the excitatory receptors at the synapses in each treatment group, though further studies are needed to fully understand this observation.

Overall, these results indicate that astrocytes pre-treated with the AChR agonist carbachol enhance the formation of functional synapses.

### Astrocyte-released TSP1 is involved in the increase in synaptic structures after cholinergic stimulation of astrocytes

We investigated whether astrocytes induce protracted expression and secretion of TSP1 after carbachol pre-treatment. Astrocytes were treated with carbachol for 24 hours followed by treatment washout and the subsequent replacement with treatment-free culture medium. We collected astrocyte medium and cells lysate at the end of the 24h incubations (t0), and 6h and 24h after treatment wash-out; levels of secreted and total cellular TSP1 were quantified by Western blot.

**Figure 6:**
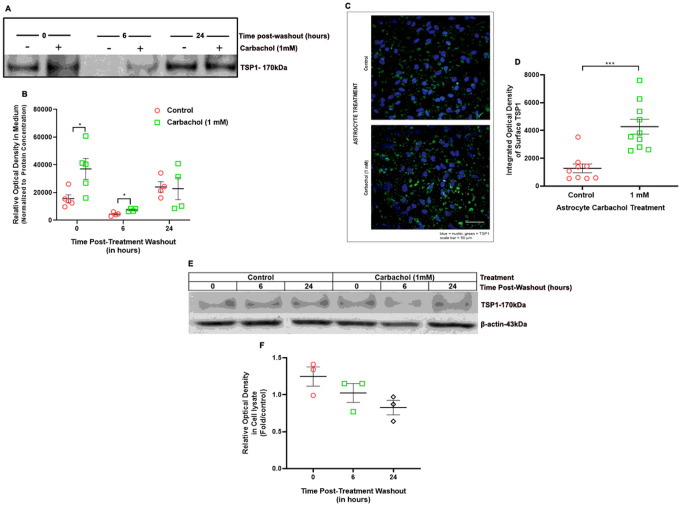
Cholinergic stimulation of astrocytes induces TSP1 release from astrocytes with no effect on TSP1 protein expression. **(A-B):** TSP1 released by astrocytes were quantified in the astrocyte-conditioned medium by Western blot. (**A**): Representative immunoblot of astrocyte conditioned medium derived from control or 1 mM carbachol-treated astrocytes immediately after 24-hour carbachol treatment (time 0) and at 6- and 24-hours post-treatment washout. (**B**): Densitometric quantification (mean ± SEM; n = 5 experiments) of astrocyte-released TSP1 normalized to total protein concentration (**C-D**): The determination of TSP1 surface levels was carried out in non-permeabilized control and carbachol-treated astrocytes 24-hours post-treatment washout by fluorescence immunohistochemistry and imaged by confocal microscopy. (**C**): Representative images of TSP1 surface expression on control (top) and carbachol (bottom) treated astrocytes 24-hours post-treatment washout. Nuclei are shown in blue; TSP1 immunofluorescence is shown in green. (**D**): Relative TSP1 fluorescence was quantified (see “Methods” for description of the unbiased quantification of immunofluorescence); (mean ± SEM; n = 9 – 10 fields from 2 coverslips). (**E-F**): TSP1 levels in astrocyte cell lysates were determined by Western blot immediately after 24-hour treatment with 1 mM carbachol (time 0) and at 6- and 24-hours post-treatment washout. (**E**): Representative immunoblot of TSP1 levels in astrocyte cell lysates (upper blot) and β-actin loading control (lower blot). (**F**): Densitometric quantification (mean ± SEM; n = 3 experiments) of astrocyte TSP1 normalized to β-actin and expressed as fold over control. Statistical analysis was performed using the Student’s t-test at each time point relative to time-matched control; *: p < 0.05; ***: p < 0.001.

Medium from astrocytes treated with carbachol showed a 2.4-fold increase in TSP1 compared to medium from untreated astrocytes at the end of the 24h incubation with carbachol (Fig. 6A, B) confirming our previous findings [51]. Six hours after replacing the treatment medium with carbachol-free medium, TSP1 was detectable, though at lower levels, suggesting continuous basal release of TSP1. Importantly, 6h after treatment wash-out, but not 24h after, the amount of TSP1 released by carbachol-treated astrocytes was still greater than that released by untreated astrocytes (Fig. 6A, B). These post-treatment time points are within the time-frame of astrocyte-neuron cocultures (see Figs. 1–3). We used immunocytochemistry and confocal imaging to measure the surface expression of TSP1 in non-permeabilized cells. Twenty-four hours after carbachol treatments, astrocytes showed a 3.35-fold increase in TSP1 surface fluorescence relative to untreated astrocytes (Fig. 6C, D), while total TSP1 levels, measured by Western blot in the astrocyte cell lysate, were not altered (Fig. 6E, F). Taken together, these results indicate that stimulation of the cholinergic pathway in astrocytes enhances the secretion of TSP1, an effect that lasts several hours after the end of carbachol treatments and could therefore be in part responsible for carbachol-treated astrocyte-induced synaptogenesis.

**Figure 7:**
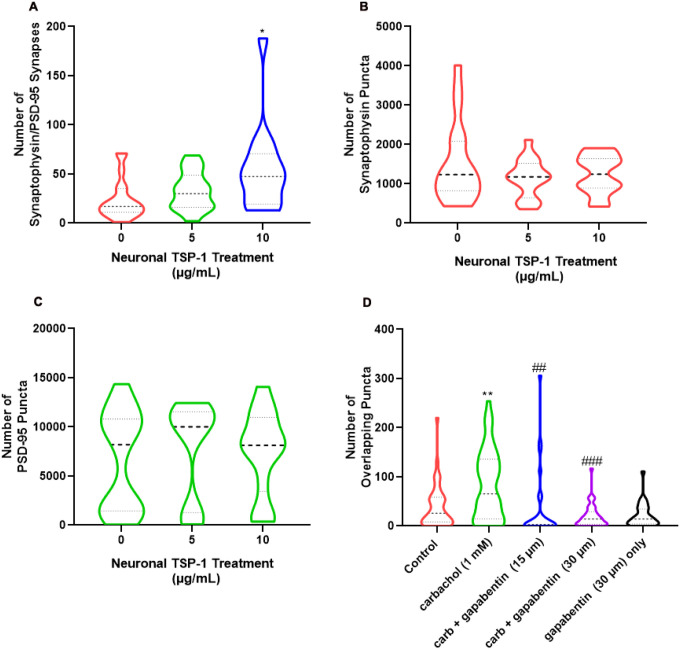
TSP1 is sufficient to increase synaptic structure formation in hippocampal neurons and TSPs are necessary to induce synaptogenesis by carbachol-treated astrocyte. **(A-C):** Primary hippocampal neurons were treated with TSP1 (5, 10 μg/mL) for 24 hours. Immunocytochemical labeling of the pre and postsynaptic proteins synaptophysin and PSD-95 was performed. Synaptophysin (**A**), PSD-95 (**B**) and overlapping pre and postsynaptic puncta (indicative of structural synapses) (**C**) were quantified and the distribution of the data is shown (the bold, dashed line represents the median; the upper and lower dotted lines represent the 75^th^ and 25^th^ percentile, respectively; n= 14–20 neurons from 5–6 coverslips). Statistical comparison of the means was performed using one-way ANOVA followed by Dunnett’s ad hoc test: *: p < 0.05 vs. control. (**D**): Astrocytes were treated for 24 hours with 1 mM carbachol or left untreated; after carbachol treatment washout astrocytes were co-cultured with hippocampal neurons for 24 hours in the presence or absence of gabapentin (15, 30 μM) to block TSP1 function in neurons. Presynaptic (synaptophysin) and postsynaptic (PSD95) puncta overlap was quantified as previously described. The distribution of the data is shown (the bold, dashed line represents the median; the upper and lower dotted lines represent the 75^th^ and 25^th^ percentile, respectively; n= 25–43 neurons from 4 independent experiments). Statistical comparison of the means was performed using one-way ANOVA followed by Bonferroni’s Multiple Comparison test. **: p < 0.01 vs. control; ##: p < 0.01, ###: p < 0.001 vs. carbachol. (A-D): The bold, dashed line represents the median; the upper and lower dotted lines represent the 75^th^ and 25^th^ percentile, respectively.

Hippocampal neurons were then directly treated with TSP1 (5 or 10 μg/mL) for 24-hours. The number of synaptophysin and PSD-95 puncta was not increased by treatment of neurons with TSP1 (Fig. 7A, B). However, the number of co-localized synaptophysin/PSD-95 puncta increased up to 2-fold relative to untreated neurons (Fig. 7C) indicating an increase in the formation of apposed pre- and postsynaptic structures.

To test whether TSP1-activated signaling in neurons is necessary for the enhanced synaptogenic effect of carbachol pre-treated astrocytes, we incubated astrocyte-neuron co-cultures with gabapentin, an inhibitor of the α2δ1 subunit of neuronal voltage-gated calcium channels that mediates the synaptogenic effects of TSPs on retinal ganglion cells [18, 52]. The addition of gabapentin (15 and 30 μM) to the co-culture system containing both neurons and carbachol pre-treated astrocytes blocked the upregulation of synaptic puncta induced by carbachol-treated astrocytes (Fig. 7C).

Together, these results indicate that TSP1, whose secretion by astrocytes is increased by carbachol treatments, is involved in the increased synaptogenesis observed after co-culture of neurons with carbachol-stimulated astrocytes.

## Discussion

Increasingly, the study of CNS function in health and disease has focused on the bi-directional communication and signaling between glia cells and neurons [10, 26, 53–58]. In particular, astrocytes have been shown to respond to external signals, including neurotransmitters and neuroactive substances [57], to modulate gene expression [59], and to release numerous factors, many of which influence synapse formation in neurons [3, 6, 8, 9, 11–14, 16, 17].

In this study we characterized the effects of cholinergic stimulation in astrocytes on the formation of active synapses. We report that cholinergic stimulation in astrocyte potentiates the formation of synapses (Figs. 1–5) in part by enhancing astrocyte secretion of the synaptogenic factor TSP1, which persists after the ACh receptor agonist has been removed (Fig. 6), and that the inhibition of TSP signaling by gabapentin abolishes the synaptogenic effects of carbachol-stimulated astrocytes (Fig. 7).

Several studies have focused on the role of ACh in brain development independent of its function as neurotransmitter; nicotinic and muscarinic ACh receptors and acetylcholinesterase are expressed in the brain before synapses are formed [43–45]. Neuronal acetylcholine release influences neuronal migration, differentiation, synapse formation and function [32, 38–42], as well as oligodendrocyte development and function [55]. However, this is the first study to report that cholinergic stimulation in astrocytes directly modulates synaptogenesis in neurons.

We found that synapse formation induced by carbachol-stimulated astrocytes is inhibited by the M3-AChR antagonist 4-DAMP (Fig. 2). We previously characterized the robust Gq-coupled signaling induced by cholinergic stimulation of muscarinic M3-AChR in astrocyte cultures [60–65]. We also identified the promoting effect of astrocyte cholinergic stimulation on neurite outgrowth in co-cultured neurons and in organotypic hippocampal cultures during early phases of neuronal maturation; these effects were mediated in part by an increased release by carbachol-stimulated astrocytes of extracellular matrix proteins laminin and fibronectin and by the modulation of the plasminogen activator extracellular matrix protease system [1, 2, 66]. Signaling through GPCRs in astrocytes is typically long-lasting because GPCRs have a slower desensitization time course than ionotropic receptors [67].

Somewhat surprisingly, the nicotinic receptor antagonist mecamylamine also attenuated the carbachol effect (Fig. 2). While ionotropic receptor signaling in neurons is usually localized and short-lived, the activation of astrocyte-expressed α7 ionotropic nAChRs has been shown to increase permeability to Ca^2+^, which in turn causes a prolonged release of Ca^2+^ from intracellular stores [23] and plays a role in synapse function by recruiting AMPA receptors to the postsynaptic surface [68].

We also found that cholinergic stimulation of astrocytes increased the frequency of mEPSCs (Fig. 5), indicating an increase in the number of functional synapses [50]. Importantly, we found that the number of puncta containing the NR2B-subunit of the NMDA receptor increased when neurons where co-cultured with ACh pre-treated astrocytes (Fig. 4). NMDARs containing this subunit are expressed during early development [69], and have been shown to be required for synaptic plasticity and synaptogenesis [70].

In a previous proteomic study we have reported that carbachol increased the release of TSP1 from astrocytes [51]; in this study, we have shown that TSP1, in the absence of astrocytes, increased synapse numbers (i.e., the colocalization of presynaptic and postsynaptic puncta), but did not increase the number of pre- or postsynaptic puncta (Fig. 7), suggesting that TSP1 may facilitate the alignment of pre and postsynaptic puncta into structural synapses. Indeed, it has been proposed that the TSPs, acting through the postsynaptic α2δ1 receptor, may exert their synaptogenic effects through the recruitment of cell adhesion and scaffolding proteins, promoting the assembly of apposing pre- and postsynaptic structural synapses [14, 17, 18, 52]. Consistent with these findings, we report that inhibition of α2δ1 signaling by gabapentin prevents carbachol-treated-astrocyte-induced increased synapses (Fig. 7). TSP1 is expressed [59] and released by astrocytes during synaptogenesis [3] and exogenous addition of TSP1 and TSP2 to retinal ganglion cells increased the number of synapses which are presynaptically active, but postsynaptically silent [3]. TSP1 also increases the speed of synapse formation in hippocampal neurons [71]. Additionally, ATP activation of astrocytes results in increased expression of TSP1 in a time and concentration-dependent manner [72].

**Figure 8:**
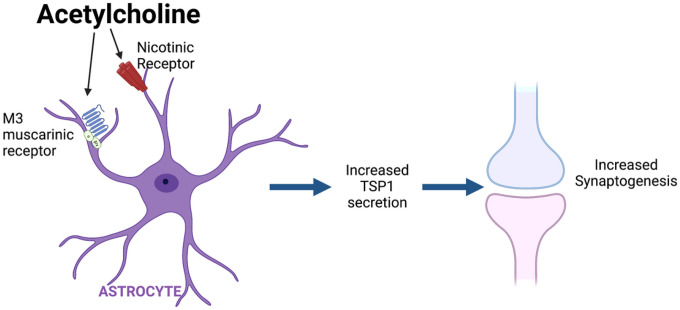
Schematic representation of the findings described in this manuscript. Cholinergic stimulation of astrocytes via M3 muscarinic and nicotinic receptors increases the formation of functional synapses. This effect is in part mediated by an increased release of TSP1 from astrocytes, which increases the alignment of pre and postsynaptic puncta into structural synapses.

In conclusion our study uncovers a new mechanism of astrocyte-neuron communication by which a signal from neurons, ACh, through the stimulation of a robust signaling pathway in astrocytes [64], leads to the increased release by these cells of synaptogenic proteins and increased formation of functional synapses. Figure 8 is a schematic representation of our findings. This study expands our understanding of the signals that regulate the release of synaptogenic proteins from astrocytes and contributes a novel mechanism by which the cholinergic system is involved in brain development.
